# A five-year risk prediction model of cardiovascular disease in individuals with bipolar disorder: a nationwide register study from Sweden

**DOI:** 10.1038/s41380-025-03381-7

**Published:** 2025-12-19

**Authors:** Maja Dobrosavljevic, Mikael Landén, Isabell Brikell, Zheng Chang, Ralf Kuja-Halkola, Paul Lichtenstein, Pontus Andell, Ole A. Andreassen, Michael Bauer, Rosa Corcoy, Giovanni de Girolamo, Andreas Reif, Henrik Larsson, Miguel Garcia‑Argibay

**Affiliations:** 1 School of Medical Sciences, Faculty of Medicine and Health, Örebro University, Örebro, Sweden; 2 Institute of Neuroscience and Physiology, Sahlgrenska Academy, Gothenburg University, Gothenburg, Sweden; 3 Department of Medical Epidemiology and Biostatistics, Karolinska Institutet, Stockholm, Sweden; 4 Department of Global Public Health and Primary Care, University of Bergen, Bergen, Norway; 5 Department of Biomedicine, Aarhus University, Aarhus, Denmark; 6 Department of Physiology and Pharmacology, Karolinska Institutet, Stockholm, Sweden; 7 ME Cardiology, Heart & Vascular Theme, Karolinska University Hospital, Stockholm, Sweden; 8 Center for Precision Psychiatry Division of Mental Health and Addiction, Oslo University Hospital & Institute of Clinical Medicine, University of Oslo, Oslo, Norway; 9 Department of Psychiatry and Psychotherapy, University Hospital Carl Gustav Carus, Medical Faculty, TUD Dresden University of Technology, Dresden, Germany; 10 Institut d’Investigació Biomèdica Sant Pau (IIB Sant Pau), Barcelona, Spain; 11 Departament de Medicina, Universitat Autònoma de Barcelona, Bellaterra, Spain; 12 CIBERBBN, Madrid, Spain; 13 IRCCS Centro San Giovanni di Dio Fatebenefratelli, Brescia, Italy; 14 Department of Psychiatry, Psychosomatic Medicine and Psychotherapy, University Hospital Frankfurt – Goethe University, Frankfurt am Main, Germany; 15 Fraunhofer Institute for Translational Medicine and Pharmacology ITMP, Frankfurt am Main, Germany; 16 Developmental Evidence synthesis, Prediction, Implementation lab, Centre for Innovation in Mental Health, Faculty of Environmental and Life Sciences, University of Southampton, Southampton, UK; 17 Hampshire and Isle of Wight NHS Foundation Trust, Southampton, UK

**Keywords:** Bipolar disorder, Predictive markers

## Abstract

Cardiovascular disease (CVD) risk prediction models for the general population may not provide accurate predictions in individuals with bipolar disorder (BD) who have elevated risks of cardiometabolic conditions and premature mortality. Therefore, we aimed to: 1) develop a five-year CVD risk prediction model in this population by using nationwide register data from Sweden, 2) investigate whether the performance improved when we considered additional risk factors, including psychiatric comorbidity, psychotropic medication, and socio-demographic variables, compared to using established CVD risk factors only, and 3) whether machine learning approach provided improvements compared to standard logistic regression models. We followed 33,933 persons with BD aged 30–82 years old, without previous CVD, from the date of BD diagnosis registered between 2007–2014, for up to five years. The logistic regression model containing only established risk factors yielded an area under the receiver operating characteristic curve (AUC) of 0.76 (95% confidence interval 0.74–0.78) in the test dataset, while the logistic regression model and the best performing machine learning model including additional predictors yielded similar results (AUC was 0.77 (0.75, 0.79) in both models). The performance of logistic regression models slightly improved with additional predictors when continuous risk scores were used. In conclusion, standard logistic regression and established CVD risk factors may be sufficient to predict CVD in individuals with BD when using population register-based data from Sweden. External validation across diverse healthcare settings and rigorous assessment of clinical impact will be crucial next steps before implementing these models in clinical practice.

## Introduction

Bipolar disorder (BD) is a chronic and severe mental disorder, characterized by alternating episodes of depression and either mania (bipolar I disorder), or hypomania (bipolar II disorder), with a global lifetime prevalence between 1–3% [1–3]. Individuals with BD have an increased risk not only of comorbid psychiatric conditions but also of a wide range of medical conditions [4], including metabolic disorders like type 2 diabetes and cardiovascular diseases (CVDs) [5–7]. Consequently, BD carries a substantial burden of disease and has been linked to premature mortality, and shorter life expectancy [7, 8]. Increased suicidality rates can partially explain the excess mortality in BD. However, about two-thirds of deaths are linked to medical conditions, with CVDs being the leading cause of premature mortality [9, 10]. It is therefore important to create risk stratification tools to identify individuals with BD who are at high risk of developing CVDs.

CVD risk stratification tools are well established for clinical use in the general population (e.g., SCORE 2, Framingham Risk Score, QRISK, PREDICT) [11–14]. These include established risk factors such as blood pressure, lipid profile, diabetes, smoking, and body mass index. Yet, these prediction models might underestimate the cardiovascular risk in the population with severe mental disorders [14–16], hence the models should be either updated within this population, or they should consider severe mental disorders as one of the predictors. To rectify this underestimation, some models have included severe mental disorders in cardiovascular risk prediction. For instance, the QRISK3, a prediction model of a ten-year cardiovascular risk in the general population aged 25 to 84 includes atypical antipsychotics and severe mental disorders along with established predictors (variables from the QRISK2) and several other additional cardiovascular risk factors [12]. Furthermore, in the PRIMROSE study, ten-year risk prediction models of the first cardiovascular event were developed specifically for people with severe mental disorders between 30 to 79 years of age, using primary care data from the UK [15, 17]. The authors found that the PRIMROSE models containing additional predictors (i.e., area-based deprivation, severe mental disorder diagnosis, prescriptions for antidepressants and/or antipsychotics, and heavy alcohol use) performed better compared to the models that included only established CVD risk factors. However, such models have not been evaluated in Sweden using available registry data nor in individuals with BD specifically, in whom CVD risk may differ from the risk in people with other severe mental disorders (e.g., schizophrenia) [8, 18].

Machine learning (ML) has emerged as a new approach in developing CVD risk prediction tools. ML may provide advantages over standard regression models by being more flexible and making fewer assumptions, e.g., using nonparametric and semiparametric methods [19]. ML algorithms can optimize the use of large-scale data and of many predictors by considering complex non-linear associations and interactions between predictors [20–22]. Several applications of ML in CVD risk prediction have shown significant improvements compared to standard regression approaches when developed in the general population [20, 22, 23]. However, such prediction models are not available for the population with BD.

In the current study, we aimed: (i) to develop and internally evaluate a five-year risk prediction model of incident CVD in people diagnosed with BD by using large-scale data from Swedish population-based registers; (ii) to investigate whether additional predictors (i.e., psychiatric comorbidity, use of psychotropic medication, and socio-demographic variables) improve the predictive accuracy of established cardiovascular risk factors available in Swedish registries (i.e., age, sex, hypertension, hyperlipidaemia, diabetes mellitus, tobacco use disorder, family history of CVD); and (iii) to compare the performance of standard logistic regression (LR) models with more complex ML models.

## Materials and methods

We followed the TRIPOD + AI statement: updated guidance for reporting clinical prediction models that use regression or machine learning methods [24]. Data management was performed using SAS software version 9.4 and statistical analyses were conducted using R V.4.3.1 (packages ‘ROCR’, ‘CalibrationCurves’, ‘rms’, ‘glmnet’, ‘caret’), and Python 3.11.9 (scikit-learn [25] version 1.5.1, imbalanced-learn [26] version 0.12.3, and XGBoost [27] version 2.0.3libraries). The study protocol is available in Supplementary material, page 18.

### Data sources

Data were acquired from a record linkage from several Swedish national registers, using the unique personal number assigned to all Swedish residents. Socio-demographic data were obtained from the Total Population Register, which contains demographic information since 1968 [28], and the Longitudinal integration database for health insurance and labour market studies register (LISA), with data such as education, income, and civils status, since 1990 on all individuals aged 16 and older [29]. We obtained information on diagnoses from the National Patient Register (NPR), containing all inpatient care diagnoses since 1987 and specialized outpatient diagnoses since 2001 [30], and the Cause of Death Register (CDR) [31], which covers all deaths from 1952. Diagnoses in the NPR and CDR are classified per the International Classification of Diseases (ICD) version 8 (1969–1986), ICD-9 (1987–1996), and ICD-10 (1997-present). Information on medication prescription was acquired from the Prescribed Drug Register (PDR) [32] which covers data on all dispensed prescriptions, with a date of prescription and dosage, since July 1st, 2005, using the Anatomical Therapeutic Classification (ATC) system. We linked individuals from our study cohort to their first-degree relatives by using the Multigeneration Register [33], to obtain information on medical family history.

### Definition of bipolar disorder

To identify individuals with BD from the NPR, we used an algorithm validated in Sweden, showing high specificity [34], which defines BD as having at least two inpatient or outpatient admissions for a core BD diagnosis (ICD-8: 296.0–296.3, 296.8, 296.9; ICD-9: 296A–296E, 296 W, 296X; and ICD-10: F30, F31), with exclusion of sole diagnoses of ICD-8: 296.2 (manic-depressive psychosis, depressed type) and/or ICD-9: 296B (unipolar affective psychosis, melancholic form). Additionally, individuals with two or more diagnoses of schizophrenia before the start of follow up were excluded from the cohort.

### Population and study period

**Fig. 1:**
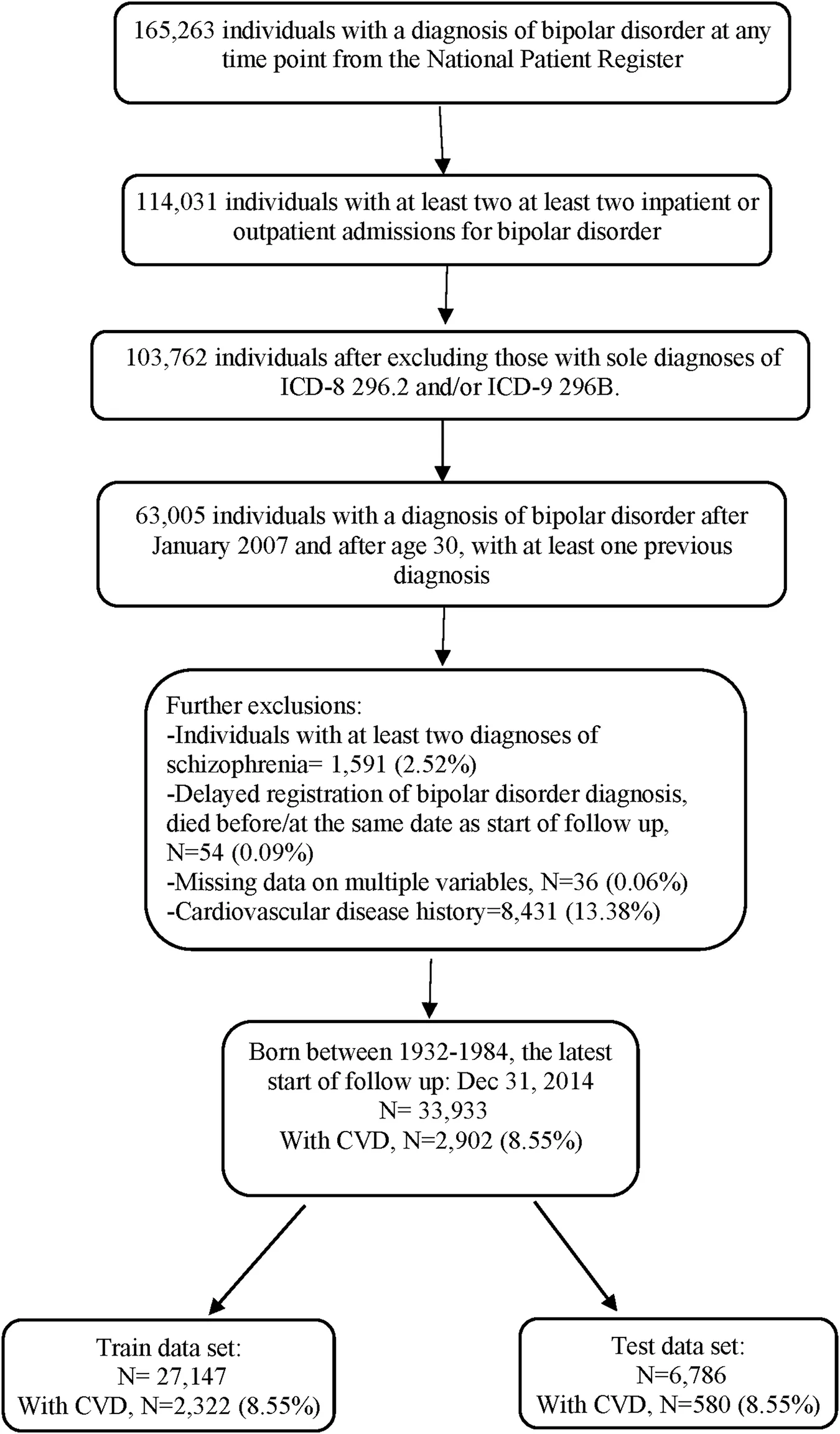
Flowchart of the study population selection process.

The study cohort included individuals with BD born between 1932–1984, without previous history of CVDs, aged 30 and older. This age cut off was selected following previous CVD risk prediction studies in people with severe mental disorders [15]. Our cohort was subsequently divided into the 80% training data set, or 20% hold-out test sample, by using a stratified random split by the outcome classes. All individuals were followed from the date of a BD diagnosis that was registered after January 1^st^, 2007, and after age 30 (i.e., first diagnosis of BD after these two dates, with at least one previously recorded diagnosis), but before December 31^st^, 2014, until diagnosis of CVD, emigration, death, or end of the five years (Fig. 1). The inclusion period started on January 1^st^, 2007, to allow for enough time for medication prescriptions to be recorded (i.e., at least 18 months from the start of PDR on July 1^st^, 2005). Although the PRIMROSE model addressed a ten-year CVD risk in people with severe mental disorders [15, 16], we chose the five-year time window as it may be more clinically appropriate for high-risk groups such as psychiatric populations.

### Candidate predictors and selection of relevant predictors

Candidate predictors were selected a priori, considering relevant literature and established CVD risk prediction models. Well-established risk prediction models for CVDs commonly consider risk factors such as blood pressure/hypertension/hypertensive therapy, body mass index (BMI), smoking, diabetes, total/LDL/HDL cholesterol [13], and family history of CVDs (i.e., first-degree relative with CVD, before age 60) [12, 13, 35, 36]. Due to the nature of available data in Swedish registers (i.e., electronic health records of diagnoses and medication prescriptions), we included proxy measures of established CVD risk factors: a diagnosis or dispensed medication prescription for hypertension, diabetes mellitus (Type I and Type II), hyperlipidaemia, obesity, and tobacco use disorder; first-degree family history (a diagnosis) of CVDs before age 60, as well as sex and age at the start of follow up (Supplementary tables 1 and 2).

We considered additional predictors as potentially relevant risk factors, including the number of psychiatric and non-psychiatric hospitalisations in the last two years, psychiatric comorbidity of BD disorders (i.e., depression, anxiety, alcohol use disorder, substance use disorder other than alcohol and tobacco, ADHD), previous use of psychotropic medication, relevant socio-demographic factors (e.g., educational attainment, birth country, civil status, having children, residential area [urban/rural] and income), and other health conditions (autoimmune diseases, migraine, epilepsy, etc.), which have been associated with CVD risk [6, 37]. Among psychotropic medications, we included the following categories as separate predictors (details in Supplementary Table 4): anxiolytics, antidepressants, hypnotics and sedatives, antiepileptics (including benzodiazepine derivates clonazepam, and other antiepileptic medication, but excluding mood stabilizers), mood stabilizers (lithium and other mood stabilizers), antipsychotics, medication for ADHD, and medication for the treatment of addictive disorders.

First, we considered a narrower set of predictors, including the lifetime history of diagnoses and medication prescriptions and most recent socio-demographic information, with multivariable LR and ML models (30 candidate predictors, Supplementary Table 1). Second, we investigated a wider set of predictors using ML models (63 predictors, Supplementary Table 2), combining lifetime history of diagnoses and recent history of medication prescriptions (within the last two years). As we did not expect that information on family medical history or socio-demographic variables was missing at random (e.g., foreign born individuals are more likely to have missing data), we created separate predictors for missingness. We also excluded individuals with missing data on multiple predictors (N = 36, or 0.06%). Details on missing data are in Supplementary Table 6.

For the standard LR approach, we applied a limited backward stepwise procedure in the training dataset to determine whether to retain candidate predictors based on their p-values. The established risk factors were kept fixed in the model, while among additional risk factors, variables with the highest p-value were sequentially rejected, until none of them remained with a p-value greater than 0.1 [38, 39]. This approach allowed us to retain established CVD risk factors in the model and to consider including additional risk factors, finally creating a relatively simple model with good face validity. All models were internally evaluated in the test dataset.

### Outcomes

We included an incident diagnosis (primary or any secondary diagnosis) of or medication prescription for the following CVDs: ischemic heart disease, cerebrovascular diseases and transient ischemic attack, thromboembolic diseases, heart failure, arteriosclerosis, and arrhythmia, acquired after the start of follow-up and within the five years. We identified individuals with CVDs based on ICD-10 diagnostic codes from the NPR and CDR. CVDs are often diagnosed in primary care, which is not covered by the NPR. We, therefore, also considered CVD dispensed medication prescriptions based on ATC codes from the PDR, which includes dispensed medication prescribed in primary care as well. The full list of ICD and ATC codes is in the Supplementary Table 5.

### Statistical analysis

For internal validation of the models, we randomly assigned individuals from the main cohort to either the 80% training data set or the 20% hold-out test sample, by using a stratified random split by the outcome classes. We first applied multivariable LR analysis to assess the associations between CVDs and candidate predictors in the training data. We also applied penalized LR which incorporates a penalty term in the loss function to address overfitting in LR models [40]. We tested LASSO (L1 regularization), Ridge (L2 regularization), and Elastic Net models (L1 and L2 regularization). A 10-fold cross-validation was used to find the best hyperparameter λ. To further investigate the robustness of our findings obtained using the backwards stepwise selection to identify relevant predictors in the LR model, we also employed the Akaike information criterion (AIC) (Supplementary material, page 17) [41]. This approach balances model fit and complexity and penalizes excessive parameters to identify relevant features.

Furthermore, we trained several ML models, including a Random Forest, XGBoost, and Histogram-Based Gradient Boosting model, which implicitly incorporate potential interactions between predictors, as well as Naïve Bayes, and Support Vector Machine. Additionally, we created a soft-voting ensemble model by combining the three top-performing models, as determined by AUC scores in the cross-validation results. To optimize the hyperparameters of each ML model, a 10-fold cross-validation with grid search was conducted, using AUC as the scoring metric. The grid including each hyperparameter space is in the Supplementary Table 8. Continuous variables were scaled for the Support Vector Machine model.

To assess the discrimination of the models, i.e., the ability of the model to differentiate those with and without CVDs, we used the receiver operating characteristic (ROC) curve with the area under the receiver operating characteristic curve (AUC) and 95% confidence intervals using the DeLong algorithm [42]. We also used the area under the precision-recall curve (AUPRC), which is a useful measure in unbalanced models (uneven class distribution in the outcome variable). Furthermore, sensitivity, specificity, positive predictive value (PPV), negative predictive value (NPV), and balanced accuracy were analysed for the predefined high-risk threshold of predicted probability set at 10 and 20% [16, 43, 44], as well as an optimal high-risk threshold calculated using the Youden’s index [45]. To assess the calibration of the model (i.e., assess the agreement between predicted and observed values at different levels of predicted probability), we used the Brier score [46], and calibration plots [47, 48]. Additionally, to test the statistical significance of the incremental value of additional predictors in LR models, we first compared the AUCs of the models with the DeLong’s test [49] and with the Net Reclassification Index (NRI), which summarises reclassification of participants when new predictors are added for high-risk thresholds of 10 and 20%. We also used two continuous measures that consider all possible thresholds of predicted probability: the category-free NRI and the Integrated Discrimination Improvement (IDI) index [50, 51].

As the cardiovascular risk differs between men and women, and the risk prominently increases on average from age 50 [52], we tested the performance of the LR models across males and females, and in those younger and older than 50 years of age.

## Results

### Descriptive information

Our study cohort comprised of 33,933 individuals with BD aged 30–82 years (mean age 47.4, SD = 12.0) at the beginning of follow-up. From the total cohort, 27,147 individuals (80%) were randomly assigned to the training dataset, of whom 2322 developed CVD (8.55%), while 6786 individuals (20%) were assigned to the hold-out or test dataset, of whom 580 (8.55%) developed CVD during the five-year follow up (Fig. 1). Descriptive characteristics are in Supplementary Table 6.

### Selected additional predictors of cardiovascular disease in logistic regression models

By using LR and the limited backwards stepwise selection procedure for predictor selection, 17 predictors were retained – nine established predictors, and eight additional risk factors (Table 1) in the training dataset. Among proxy measures of established risk factors, the strongest association with CVDs was found for diagnosis or medication prescription for hypertension (OR = 1.57, 95% CI 1.39–1.76, p < 0.001). Among additional risk factors, substance use disorder other than tobacco and alcohol (OR = 1.37, 1.20–1.57, p < 0.001) and antiepileptic medication (OR = 1.37, 1.14–1.64, p < 0.001) had the strongest association with CVDs. Majority of antiepileptic medication prescriptions (53%) was for benzodiazepine derivate clonazepam (ATC code N03AE01).

**Table 1:** Associations between the retained predictors and cardiovascular diseases for the standard logistic regression approach as odds ratios (OR) with 95% confidence intervals (CI).

	Established risk factors	OR (95% CI)
1	Age	1.07 (1.07, 1.07)***
2	Male sex	1.45 (1.33, 1.59)***
3	Hypertension	1.57 (1.39, 1.76)***
4	Diabetes mellitus	1.23 (1.04, 1.45)*
5	Hyperlipidemia	1.03 (0.89, 1.20)
6	Obesity	1.49 (1.20, 1.85)***
7	Smoking	1.35 (1.10, 1.66)**
8	Family history: present	1.21 (1.08, 1.35)***
9	Family history: missing data	1.24 (1.04, 1.47)*
	**Additional risk factors**	
10	Substance use disorder other than tobacco and alcohol	1.37 (1.20, 1.57)***
11	Sleep disorders	1.09 (0.99, 1.20).
12	Anxiolytics	1.11 (1.01, 1.22)*
13	Antiepileptics	1.37 (1.14, 1.64)***
14	Antipsychotics	1.13 (1.03, 1.24)**
15	The number of hospitalizations in the last two years	1.06 (1.04, 1.08)***
16	Low education	1.16 (1.04, 1.29)**
17	Non-Swedish country of birth	0.77 (0.64, 0.93)**

Significance codes: 0 ‘***’ 0.001 ‘**’ 0.01 ‘*’ 0.05 ‘.’ 0.1 ‘ ’.

### Model performance measures and incremental value of additional risk factors in logistic regression models

**Fig. 2:**
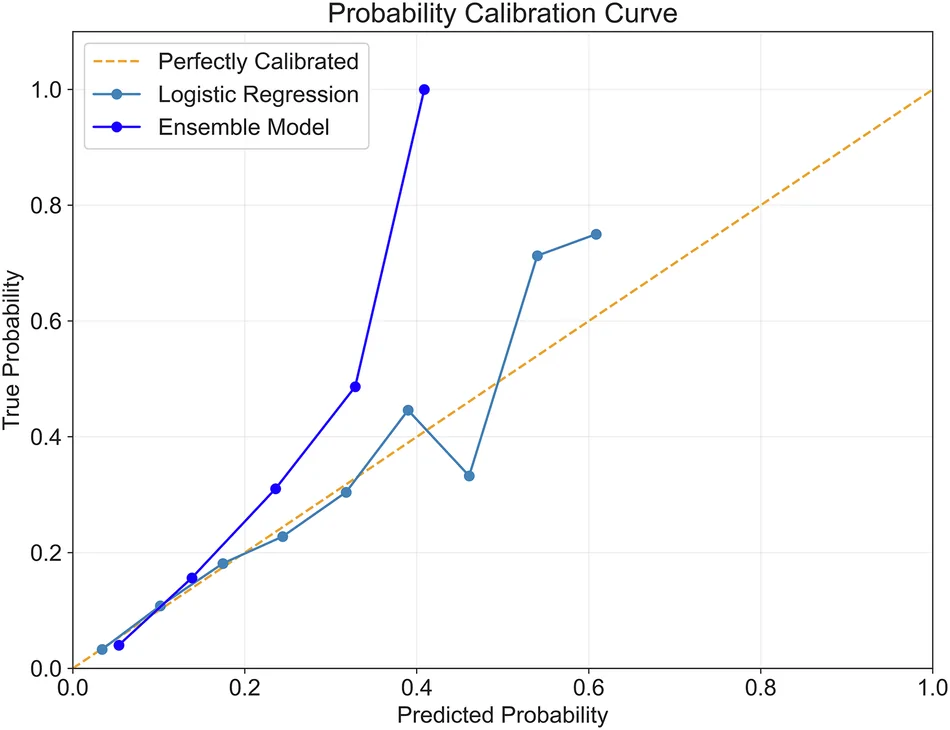
Calibration curve for logistic regression including established and novel predictors (17 predictors) and ensemble model (63 predictors) in the test dataset.

We first tested the performance of the standard LR model, which included nine established risk factors. In the test data set, the model showed an AUC of 0.76 (0.74–0.78), an AUPRC of 0.25 and a Brier score of 0.07 (Table 2), suggesting good model discrimination and calibration. The LR model containing additional eight predictors (17 predictors in total) showed a similar AUC of 0.77 (0.75–0.79) in the test data set, with an AUPRC of 0.25 (see Table 2, and Supplementary fig. 1 for corresponding ROC and AUPR curve), and a Brier score of 0.07 (calibration curve is in Fig. 2). Penalized LR provided results consistent with the standard LR analysis (Table 2). We compared the performance of the LR model containing only established risk factors versus the model containing additional risk factors using the DeLong test, which showed that the difference in AUC between the two models was not statistically significant (p value 0.296). Furthermore, the model derived based on the AIC criteria yielded results consistent with the model derived based on the stepwise selection process, albeit this model kept only four additional predictors (Supplementary material, page 17).

**Table 2:** Performance measures for different trained models.

Model	AUC with 95% CI		AUPRC Test dataset	Brier score Test dataset
	Train dataset	Test dataset		
Model with established risk factors only				
Logistic regression	0.747 (0.737–0.758)	0.765 (0.745–0.785)	0.248	0.071
Models with additional predictors (17 predictors)^1^				
Logistic regression	0.757 (0.747–0.767)	0.768 (0.749–0.788)	0.255	0.071
Penalized logistic regression^2^	0.757 (0.747–0.767)	0.768 (0.749–0.788)	0.254	0.071
Random forest	0.753 (0.742, 0.763)	0.760 (0.740, 0.780)	0.242	0.073
Xgboost	0.875 (0.866, 0.883)	0.723 (0.703, 0.743)	0.175	0.076
Naïve Bayes	0.715 (0.705, 0.725)	0.721 (0.701, 0.742)	0.188	0.140
Support Vector Machine	0.703 (0.691, 0.715)	0.565 (0.537, 0.593)	0.139	0.078
Histogram-Based Gradient Boosting	0.773 (0.763, 0.783)	0.761 (0.741, 0.780)	0.228	0.073
Ensemble^3^	0.782 (0.772, 0.792)	0.767 (0.747, 0.786)	0.249	0.072
Broader set of predictors (63 predictors)				
Logistic regression	0.762 (0.752, 0.772)	0.767 (0.747, 0.786)	0.251	0.071
Random forest	0.810 (0.801, 0.819)	0.763 (0.743, 0.783)	0.2437	0.072
Xgboost	0.971 (0.968, 0.975)	0.721 (0.701, 0.742)	0.181	0.075
Naïve Bayes	0.699 (0.688, 0.710)	0.709 (0.687, 0.731)	0.205	0.173
Support Vector Machine	0.956 (0.950, 0.961)	0.640 (0.615, 0.666)	0.1738	0.076
Histogram-Based Gradient Boosting	0.778 (0.769, 0.788)	0.762 (0.742, 0.782)	0.2339	0.073
Ensemble^3^	0.790 (0.781, 0.799)	0.770 (0.751, 0.789)	0.256	0.072

^1^Established risk factors plus additional risk factors, ^2^Best lasso model selected among LASSO (L1 regularization), Ridge (L2 regularization), and Elastic Net models, ^3^The ensemble model is comprised of a random forest, histogram-based gradient boosting, and logistic regression.

*AUC* area under the receiver operating characteristic curve, *AUPRC* area under the precision recall curve.

We tested several high-risk thresholds including the two predefined thresholds of 10 and 20%, as well as an “optimal” threshold obtained using the Youden’s index from the LR model (9% for both LR models). Using the 10% high-risk threshold in the test dataset, which was closely aligned with the calculated optimal high-risk threshold, the model showed a sensitivity of 0.67 (0.63, 0.70), specificity of 0.74 (0.73, 0.75), PPV of 0.19 (0.18, 0.21), NPV of 0.96 (0.95, 0.96) and balanced accuracy of 0.70 (Table 3). We did not find a statistically significant improvement when we compared the performance of the models using a high-risk threshold of 10%, with the NRI being −0.001 (95% CI −0.024, 0.021), or 20%, the NRI was 0.013 (95% CI −0.011, 0.036). Yet, we identified a statistically significant improvement when we used category-free or continuous measures (the IDI index was 0.006, 95% CI 0.003, 0.009, and integrated NRI was 0.006, 95% CI 0.003, 0.009).

**Table 3:** Sensitivity, specificity, positive predictive value (PPV), negative predictive value (NPV) and balanced accuracy for prespecified high-risk thresholds for a logistic regression model containing established risk factors only (Old model), a logistic regression model containing both established and additional risk factors (New model), and ensemble model in the test dataset.

Threshold		Sensitivity	Specificity	PPV	NPV	Balanced accuracy
0.1/10%	Old model	0.67 (0.63, 0.71)	0.74 (0.73, 0.75)	0.19 (0.18, 0.21)	0.96 (0.95, 0.97)	0.704
	New model	0.67 (0.63, 0.70)	0.74 (0.73, 0.75)	0.19 (0.18, 0.21)	0.96 (0.95, 0.96)	0.704
	Ensemble model^*^	0.65 (0.61, 0.69)	0.75 (0.73, 0.76)	0.19 (0.18, 0.21)	0.96 (0.95, 0.96)	0.697
0.2/20%	Old model	0.29 (0.26, 0.33)	0.93 (0.92, 0.93)	0.27 (0.24, 0.31)	0.93 (0.93, 0.94)	0.610
	New model	0.31 (0.27, 0.35)	0.92 (0.92, 0.93)	0.28 (0.24, 0.31)	0.93 (0.93, 0.94)	0.617
	Ensemble model^*^	0.23 (0.20, 0.26)	0.96 (0.95, 0.96)	0.33 (0.29, 0.38)	0.93 (0.92, 0.94)	0.593

^*^Ensemble model includes all 63 predictors.

LR models performed similarly across males and females (Supplementary Table 9) as in the total test dataset. However, their performance was weaker when it was tested separately in individuals younger than 50, and older than 50 years. To further investigate differences in CVD risk prediction between these two age groups, we performed a post hoc analysis where we retrained the logistic regression model with established risk factors separately within each group (Supplementary material, page 16). This analysis did not reveal improvements in the re-trained models. Moreover, predictor coefficients remained generally similar between the models in younger and older individuals, except for obesity, which appeared to be more relevant for CVD risk in those younger than 50.

### Machine learning models compared to the standard logistic regression approach

When evaluating the performance of the tuned ML models trained with 17 predictors, all showed lower AUROC and AUPRC values on the test set compared to LR, except for the ensemble model (comprising a random forest, histogram-based gradient boosting, and LR), which demonstrated comparable performance (AUC of 0.77 vs 0.77; AUPRC of 0.25 vs 0.26) (Table 2). However, when a larger set of predictors was considered, the ensemble model outperformed LR minimally, with an AUC of 0.77 versus 0.77 and an AUPRC of 0.26 versus 0.25. The balanced accuracy of the ensemble model was 0.70 with a high-risk threshold of 10% and 0.59 with a threshold of 20%, comparable with LR models (Table 3). Furthermore, with a high-risk threshold of 20%, the ensemble model outperformed the LR model when it comes to specificity (0.96 vs 0.92) and PPV (0.33 vs 0.28), although sensitivity (0.23 vs 0.31) was somewhat lower compared with LR.

## Discussion

We used registry-based data from Sweden to develop and internally validate a five-year risk prediction model of CVD in people with BD. We found that established cardiovascular risk factors and a standard LR approach provided relatively good predictive performance, with an AUC of 0.76 in the test dataset. Additional predictors and ML methods did not provide substantial improvements. As there are no available CVD risk prediction models in people with BD, we compared the achieved AUC to established cardiovascular risk prediction models, developed and validated in the general population [53], and models developed in people with severe mental disorders [15]. These models, which have demonstrated promising results for clinical application, yielded similar results to ours [17].

We observed no improvement in AUC with additional predictors, whether with the narrower (17 predictors) or broader set of predictors (63 predictors). This finding held for both the LR model and the best-performing ensemble ML models, with both achieving an AUC of 0.77. Thus, our study confirmed the relevance of established cardiovascular risk factors in the population with BD, even when they are defined as registry-based data available in Swedish electronic health records, used as proxy measures of detailed clinical indicators. The only non-significant association of CVD was with hyperlipidaemia, defined as either a diagnosis or dispensed medication prescription for hyperlipidaemia. This could be due to a potential underdiagnosis of the condition or successful remission following the treatment. The relatively modest performance of LR models with additional predictors and ML approaches compared to LR with established risk factors only, likely stems from several interconnected factors. First, additional predictors are highly co-occurring with the established risk factors. Thus, established risk factors may provide an approximation of the CVD risk that incorporates the effects of additional predictors as well. For instance, socio-demographic factors and psychiatric comorbidity may be reflected through health-adverse lifestyle choices (e.g., lack of exercise, inadequate diet, etc), which in turn, affect established risk factors. Second, the binary nature of most predictors (i.e., presence/absence of diagnoses and medication use) might have reduced the opportunity for ML to discover complex, non-linear associations and interactions between predictors. This is in line with previous ML studies on the prediction of major chronic diseases with low incidence (e.g., CVDs, chronic kidney disease, diabetes, and hypertension) and predominantly linear associations with simple clinical predictors [54]. Conversely, the advantages of ML models become more pronounced when analysing high-dimensional data with complex interactions, such as continuous physiological, psychosocial and environmental factors, in addition to medical history of diagnoses/medication prescriptions [20, 22, 23]. Finally, the modest number of events relative to the number of predictors may have limited the ability of ML to learn more complex patterns without risking overfitting, making the more parsimonious logistic regression approach equally effective.

Nevertheless, when continuous risk prediction scores were applied in LR models, eight additional predictors provided a statistically significant increment compared to established risk factors only. This finding supports previous research indicating increased cardiovascular risks associated with the use of psychotropic medication [55], or the risk associated with psychiatric conditions treated with these medications (i.e., anxiety, sleep disorders) [56–58]. Furthermore, previous research has identified that individuals with severe mental disorders and substance use disorder are at even higher CVD risk than those without substance abuse [59, 60]. Moreover, patients with BD have high rates of psychiatric comorbidity, with a concurrent use of several psychotropic medications (i.e., polypharmacy) [61], and psychiatric polypharmacy has been associated with risks of cardiovascular morbidity and mortality [62]. We did not find that mood stabilizers (including lithium and other mood stabilizers) have a statistically significant association with CVDs. This may be due to the low variability of this predictor in our cohort (i.e., 72% of the cohort had a prescription for mood stabilizers). Further, socio-economic factors (low education) were relevant for CVD risk prediction [63, 64], indicating that patients with lower education may need additional support to adequately manage cardiometabolic risk factor (e.g., diet, exercise, smoking). We also found that being born outside Sweden was associated with a decreased risk of CVD, while having missing information on family history was associated with an increased risk of CVD. These findings seem contradictory as foreign-born individuals were more likely to have missing data for family history. The cardiovascular health of immigrants compared to Swedish-born individuals may depend on their country of origin, reasons for/duration of immigration, or the selection effect [65], while also less severe CVDs may be underdiagnosed/under-reported in immigrants with mental disorders. As we did not consider more detailed information on immigration or other potential reasons for data missingness, it is difficult to translate these findings into specific clinical implications.

All tested models performed better with a high-risk threshold of 10% compared to 20% for sensitivity and balanced accuracy, a useful measure of predictive performance for unbalanced data [66]. This result may be due to the lower thresholds likely reflecting the true probability of the outcome (the mean predicted probability in the test data was 8%, and the incidence of CVD within the follow up period was 8.5%), and the calculated optimal high-risk thresholds being close to 10%. Also, this finding supports the previously used high-risk threshold of 10% in the general population for a five-year CVD risk prediction [44], and the NICE guidelines recommendations of using a 10% threshold for the initiation of preventative therapy with statins [67]. In our study, when a high-risk threshold of 10% was applied, the LR model containing both established and additional risk factors correctly classified 388 out of 580 persons with CVD at the end of follow up (67%), while with the high-risk threshold set at 20%, the same model correctly classified 180 individuals (31%). Due to the unavailability of CVD risk prediction models in people with BD, we could only compare our results with the PRIMROSE models [15]. The PRIMROSE BMI model correctly classified 390 out of 2096 men who developed CVDs (18.6%) as being high-risk, while the PRIMROSE lipid model correctly classified 410 out of 2137 men (19.2%), with similar results for women. However, the PRIMROSE models only applied a 20% high-risk threshold in calculating a 10-year CVD risk in people with severe mental disorders and incorporated both detailed continuous risk factors and history of diagnoses/medication prescriptions.

With regards to the potential clinical implications of our findings, the derived LR models, containing predominantly lifetime history of risk factors, could be applied during a single psychiatric visit in individuals with BD aged 30 and above. The ML models, incorporating a broader set of predictors with both lifetime history of diagnoses and recent history of medication prescriptions, may be more useful for follow-up assessments. Nevertheless, the derived models might need to be updated using more detailed measures from regular follow-up visits to provide adequate repeated risk calculations. Further, although our results confirmed the relevance of established risk factors for CVD in individuals with BD, eight additional risk predictors might provide improvements in predictive accuracy when continuous risk scores are applied. The use of eight additional predictors in LR models would not demand substantial additional clinical resources as these data often routinely collected in psychiatric care. Our results also emphasize the need of not only close monitoring of general health, including established risk factors, but also of psychiatric comorbidity, psychotropic medication use, and socio-economic factors to enable timely and adequate prevention of CVDs in this population. This, albeit small, increment may still be clinically relevant as it has been shown that patients with psychiatric disorders can experience poorer management of cardiovascular risk compared to the general population [68].

### Strengths and limitations

In this study we used large-scale data with national coverage of medical records and socio-demographic information in Sweden, and the considered predictors could be collected either during a clinical interview or from medical records. The investigated models, both standard LR and ML models, showed good discrimination. Furthermore, although considered predictors are clinically relevant indicators of CVD risk factors (e.g., hypertension, hyperlipidaemia, diabetes), preclinical stages and continuous measures of risk factors (e.g., cardiometabolic risk indicators such as blood pressure, glucose and lipid levels) may provide more detailed predictions of the outcomes. We also only had access to data from specialist care, while additional access to primary care records could provide a wider coverage including individuals with less severe clinical presentations of considered conditions. This limitation is reflected in our observed underestimation of the population prevalence of obesity and smoking in Sweden [69, 70]. Finally, we did not have access to other relevant CVD risk factors, such as physical activity and nutrition [71]. Thus, obtained models require further evaluation and updating by using additional data sources (e.g., primary care) and more detailed measures of risk factors. Such measures could be potentially obtained from Swedish quality registers, such as the Swedish National Diabetes Register (NDR) that provides a possibility to investigate cardiometabolic conditions registered in the primary and specialist health care, while the Swedish National Quality Register for Bipolar Disorder – BipoläR provides detailed clinical data on individuals with BD.

Furthermore, our models demonstrated reduced performance when tested separately in individuals younger and older than 50, and this did not improve when the model with established risk factors was retrained separately in these two age groups. Regarding individual predictor relevance, only obesity appeared to be more relevant for CVD risk prediction in younger individuals compared to older individuals, while other predictors showed similar relevance across both groups. Therefore, potential effects of age warrant further investigation.

Finally, in our LR modelling strategy, we employed a limited backward stepwise selection for additional risk factors while retaining all established risk factors in the model. This strategy served two purposes: maintaining clinical relevance by including established cardiovascular predictors and identifying additional important factors while preserving face validity for clinicians. To address potential overfitting concerns inherent in the stepwise selection [72], we evaluated our model in a hold-out test dataset. We also employed the AIC approach to select relevant predictors among additional risk factors and conducted a penalized LR analysis to evaluate the performance of the simple LR model with additional predictors selected in the stepwise selection approach. These analyses provided performance metrics consistent with the main findings. This is probably because we have a relatively large data set, and a modest amount of candidate predictors (30 candidate predictors) relative to the number of outcomes (580 individuals in test data). Nevertheless, to ensure the broader applicability of our findings, external validation across different healthcare systems, countries, and data sources remains essential. This validation will be particularly important given the potential variations in healthcare and documentation practices (e.g., without access to universal health care and/or to electronic health records), and patient populations across different settings.

## Conclusions

Our study suggests that standard LR using established CVD risk factors achieved satisfactory predictive performance for five-year CVD risk in individuals with BD using Swedish register-based data. This parsimonious approach proved effective, while incorporating additional predictors, i.e., psychiatric comorbidity, use of psychotropic medication and socio-demographic factors, may only provide modest improvements when continuous risk scores are used. Further studies are needed to consider more detailed health-related variables in this population (e.g., continuous measures of cardiometabolic risk, physical activity, and nutrition) to confirm or update our findings. External validation across diverse healthcare settings and rigorous assessment of clinical impact will be crucial next steps before implementing these models in routine clinical practice.

## Supplementary information

Supplementary material

## Data Availability

The Public Access to Information and Secrecy Act in Sweden prohibits us from making individual level data publicly available. Researchers who are interested in replicating our work can apply for individual level data at Statistics Sweden: www.scb.se/en/services/guidance-for-researchers-and-universities/. The code can be made available upon request.

## Code availability

The Public Access to Information and Secrecy Act in Sweden prohibits us from making individual level data publicly available. Researchers who are interested in replicating our work can apply for individual level data at Statistics Sweden: www.scb.se/en/services/guidance-for-researchers-and-universities/. The code can be made available upon request.
