# Supplementary material for: A five-year risk prediction model of cardiovascular disease in individuals with bipolar disorder: a nationwide register study from Sweden
Source: Mol Psychiatry. 2025 Dec 19;31(5):2489–97. doi: 10.1038/s41380-025-03381-7 (PMC13099429; doi:10.1038/s41380-025-03381-7)
Supplement: Supplementary file 1 — Supplementary material [file 41380_2025_3381_MOESM1_ESM.docx]

**Supplementary material**

**A five-year risk prediction model of cardiovascular disease in individuals with bipolar disorder: a nationwide register study from Sweden**

Maja Dobrosavljevic, Mikael Landén, Isabell Brikell, Zheng Chang, Ralf Kuja-Halkola, Paul Lichtenstein, Pontus Andell, Ole A. Andreassen, Michael Bauer, Rosa Corcoy, Giovanni de Girolamo, Andreas Reif, Henrik Larsson, Miguel Garcia‑Argibay

**Supplementary Table 1.** Candidate predictors: standard logistic regression model ***Page 2***

**Supplementary Table 2.** Broad set of candidate predictors of cardiovascular disease in people with bipolar disorder for machine learning models ***Page 4***

**Supplementary Table 3.** ICD-8/9/10 for health-related variables and ATC codes for corresponding medication prescriptions ***Page 6***

**Supplementary Table 4.** Groups of medication prescriptions with ATC codes ***Page 8***

**Supplementary Table 5.** List of cardiovascular diseases considered as outcomes (ICD-10) and predictors (1st-degree family history, ICD-8/9/10) and corresponding ICD codes ***Page 9***

**Supplementary Table 6.** Baseline characteristics of the study populations regarding the candidate predictors in the logistic regression model ***Page 10***

**Supplementary Table 7.** Characteristics of the study population in the train and test datasets regarding the frequency of the outcome cardiovascular disease (CVD). ***Page 12***

**Supplementary Table 8.** Grid-search parameter space for each trained model. ***Page 13***

Backwards stepwise procedure of additional predictors selection for the logistic regression model ***Page 14***

**Supplementary Figure 1.** a) ROC curve in testing data for logistic regression, b) AURP curve in testing data for logistic regression. ***Page 15***

**Supplementary Table 9.** Performance of logistic regression containing established risk factors only (Old model) and the derived logistic regression model with additional risk factors (New model) across different subgroups in the test dataset ***Page 15***

**Post hoc analyses**

1. Performance of the simple logistic regression model with established risk factors separately in individuals younger than 50, and aged 50 and older

***Page 16***

1. Application of the Akaike information criterion (AIC) to select relevant predictors ***Page 17***

**Study Protocol *Page 18***

**Supplementary Table 1.** Candidate predictors: standard logistic regression model

| **Established risk factors** | | | **Additional risk factors** | | |
| --- | --- | --- | --- | --- | --- |
|  | **Variable** | **Data source and type** |  | **Variable** | **Data source and type** |
| 1 | Age at the start of follow-up | Total population register, continuous | 10 | Anxiety diagnosis | National patient register, Binary, ICD-8,9,10 codes  -Lifetime history |
| 2 | Sex | Total population register, binary (male/female) | 11 | Depression diagnosis |  |
| 3 | Hypertensive diseases (diagnosis or medication prescription) | National patient register, Binary, ICD-8,9,10 codes, and  Prescribed drug register, Binary, ATC codes  -Lifetime history | 12 | Attention deficit hyperactivity disorder diagnosis |  |
| 4 | Type 1 or type 2 diabetes (diagnosis or medication prescription) |  | 13 | Substance use disorder, other than tobacco and alcohol diagnosis |  |
|  |  |  | 14 | Alcohol use disorder diagnosis |  |
|  |  |  | 15 | Sleep disorders diagnosis or prescription for sleep disorder medication |  |
| 5 | Hyperlipidemia (diagnosis or medication prescription) |  |  |  |  |
|  |  |  | 16 | Number of hospitalizations | Continuous  -Within the last 2 years |
|  |  |  | 17-24 | Psychotropic medication (entered as separate variables) * | Prescribed Drug Register, binary (ATC codes)  -Lifetime history |
| 6 | Obesity (diagnosis) |  | 25-26 | Educational attainment  (Educational level and Missing information on education level are entered as separate variables) | Binary, Secondary and higher education (0): secondary, post-secondary/university education; and low (1): in/complete elementary school (9 year or less)  -Most recent available information |
| 7 | Smoking (diagnosis of tobacco use disorder or dispensed medication prescription for smoking cessation) |  |  |  |  |
|  |  |  | 27 | Personal disposable income** | Negative (in debt)  Zero  Low (<20th percentile)  Medium (20th – 80th percentile)  High (>80th percentile)  -Most recent available information |
|  |  |  | 28 | Birth country | Total population register  Born in Sweden/not born in Sweden |
| 8  9 | Family history of CVD  (first-degree relatives before age 60)  Missing information of family history |  |  |  |  |
|  |  |  | 29 | Area of residence (urbanicity) | 2 levels  1- Large city region, 2 - Dense region near large city, 3 - Dense region remotely located,-as urban, and  4 - Rural region near large city, 5 - Rural region remotely located, 6 - Rural region very remotely located, as rural areas  -Most recent available information |
|  |  |  | 30 | Civil status (not married/no registered partner) | Yes (single): if surviving partner/ unmarried/ divorced/divorced partner/widow/er  No: if married/ /registered partner  -Most recent available information |

Time window for health-related variables: any history/lifetime history of diagnosis or medication prescription, for socio-economic variables: the most recent available data was retrieved.

*17) Anxiolytics, 18) Hypnotics and sedatives except for medication used for treatment of sleep disorders, 19) Antiepileptics, 20) Antidepressants, 21) Mood stabilizers, 22) Antipsychotics, 23) Medication used for the treatment of ADHD, 24) Drugs used for addictive disorders (alcohol and opioid dependance) (detailed ATC codes in Supplementary table 4)

**Personal disposable income was standardized in accordance with population distribution for the corresponding year

**Supplementary Table 2.** Broad set of candidate predictors of cardiovascular disease (CVD) in people with bipolar disorder for machine learning models

*Note: Health-related predictors, diagnoses and medications prescriptions are binary variables, only the number of hospitalizations is continuous. Total number of predictors is 63, after coding data missingness for family history of CVD in parents and full siblings, death of a parent before age 60, education, income, and having children with different partners, as separate predictors.*

|  | **Health related predictors: diagnoses^1^** | |
| --- | --- | --- |
| 1 | Hypertension | |
| 2 | Type 1 diabetes | |
| 3 | Type 2 diabetes | |
| 4 | Hyperlipidemia | |
| 5 | Obesity | |
| 6 | Tobacco use disorder | |
| 7 | Family history of CVD (1^st^ degree relative, by age 60) parents | |
| 8 | Family history of CVD (1^st^ degree relative, by age 60) full siblings | |
| 9 | Death of a parent due to a cause other than CVD before age 60 | |
| 10 | Dementia diagnosis | |
| 11 | Epilepsy | |
| 12 | Migraine | |
| 13 | Arthritis/osteoarthritis/joint pain | |
| 14 | Autoimmune diseases | |
| 15 | Anxiety disorders | |
| 16 | Depressive disorders | |
| 17 | Attention deficit/hyperactivity disorder | |
| 18 | Substance use disorder other than tobacco and alcohol | |
| 19 | Sleep disorders | |
| 20 | Alcohol use disorder | |
| 21 | Intellectual disability | |
| 22 | Pervasive developmental disorders | |
| 23 | Personality disorders | |
| 24 | Eating disorders | |
| 25 | Family history (1^st^ degree relative) of psychiatric illness | |
| 26 | Number of nonpsychiatric hospitalizations within last 2 years | |
| 27 | Number of psychiatric hospitalizations within last 2 years | |
|  | **Health related predictors: Medication prescriptions^2^** | |
| 1 | Hypertension medication prescription | |
| 2 | Medication for treatment of diabetes | |
| 3 | Hyperlipidemia medication prescription | |
| 4 | Medication prescription for smoking cessation | |
| 5 | Sleep disorders medication | |
| 6 | Corticosteroids | |
| 7 | Alzheimer’s disease medication prescription | |
| 8 | Anxiolytics | |
| 9 | Hypnotics and sedatives (except medication for sleep disorders) | |
| 10 | Antiepileptics | |
| 11 | Antidepressants | |
| 12 | Mood stabilizers | |
| 13 | Antipsychotics | |
| 14 | Medication used for the treatment of ADHD | |
| 15 | Drugs used for addictive disorders (alcohol and opioid dependance) | |
|  | **Sociodemographic variables^3^** | **Description/definition** |
| 1 | Age at the start of follow-up | -continuous variable |
| 2 | Male sex | -Binary |
| 3 | Educational attainment (2 dummy variables) | -Categorical - 3 levels: <=9 years/10-12 years/>12 years |
| 4 | Income^4^ (4 dummy variables) | -Categorical – 5 levels: Negative (in debt)/Zero/Low (<20th percentile)/Medium (20th – 80th percentile)/ High (>80th percentile) |
| 5 | Birth country | -Binary:  1= Being foreign born (in a country other than Sweden)  0= Born in Sweden |
| 6 | Area of residence | -Binary  Urban: Large cities and commuting municipalities near large cities; Medium-sized towns and commuting municipalities near medium-sized towns; Smaller towns and commuting municipalities near smaller towns  Rural: Rural municipalities with a population of less than 15 000, very low commuting rate (less than 30 %); Rural municipalities with a visitor industry. |
| 7 | Civil status | -Binary:  1= Surviving partner/ unmarried/ divorced/divorced partner/widow/er  0= Married/ /registered partner |
| 8 | -Having children | -Binary |
| 9 | -Having children with different partners | -Binary |

^1^Time window for diagnoses: any history prior to follow-up start

^2^Time window for medication prescriptions is within 2 years prior to follow-up start

^3^Time window for SES variables is set in the year with available information closest (prior) to the follow-up start.

^4^ Personal disposable income was standardized in accordance with population distribution for the corresponding year

**Supplementary Table 3.** ICD-8/9/10 for health-related variables and the Anatomical Therapeutic Classification system (ATC) codes for corresponding medication prescriptions

|  | **ICD-8** | **ICD-9** | **ICD-10** | **ATC** |
| --- | --- | --- | --- | --- |
| Hypertensive diseases (diagnosis and medication prescription) | 400-404 | 401-405 | I1 | C03A, C08C, C09A, C09B, C09C |
| Type 1 diabetes (diagnosis and medication prescription) | 250 | 250 | E10 | A10 (any) |
| Type 2 diabetes (diagnosis and medication prescription) | 250 | 250 | E11 |  |
| Obesity (diagnosis) | 277 | 278A, 278B | E65-E66 | - |
| Hyperlipidemia | 279 | 272 | E78 | C10 |
| Smoking (tobacco use disorder diagnosis and medication for smoking cessation) | _ | - | F17 | N07BA01, N07BA03 |
| Alzheimer’s disease and other dementias diagnosis, and medication used for treatment of Alzheimer’s disease | 290, 2930, 2931 | 290A, 290B, 290E, 290W, 290X, 294B, 331B, 331A, 331C, 331X | G30, G31.1, G31.8, F00-F03, F05.1 | N06D |
| Epilepsy | 345 | 345 | G40-41 | - |
| Migraine | 34609 | 346A/B/X/W | G43 | - |
| Arthritis/osteoarthritis/joint pain (diagnosis) | - | - | M0, M1, M2, R26 | - |
| Autoimmune disease – any (diagnosis) | 0341 13607 24200 24503 25810 26910 28700 28710 340 35401 390 391 392 44609 44630 44638 44640 56300 56310 57190 580 582 694 69610 69619 69620 69621 69622 69623 70400 71200 71210 71239 71600 73300 73400 73410 | 034B 136B 242A 245C 258B 287A 287D 340 357A 358A 390 391 392 446A 446B 446F 446G 555 556 571F 579A 580 582 694A 694E 694F 696 704A 710A 710B 710C 710D 710W 714A 725 | A389 D686 D690 D693 E050 E063 E310 G04 G131 G35 G610 G700 I00 I01 I02 L100 L120 L13 L40 L63 K900 K50 K51 K743 M06 M300 M301 M303 M311 M315 M317 M32 M339 M34 M350 M351 M352 M353 N00 N01 N03 N05 | - |
| Sleep disorders (diagnosis and medication prescription) | 306.40 | 307E, 780F | G47, F51 | N05CH01, N05CF01, N05CF02, N05CF03 |
| Anxiety disorders | 300 (except 300.4) | 300 (except 300E) | F40-F42, F45 |  |
| Depression | 2962.x, 3004.x | 296B.x, 300E.x, 311 | F32.x, F33.x, F34.x, F38 F39 |  |
| Attention-deficit/hyperactivity disorder | _ | 314 | F90 |  |
| Bipolar disorder | 296.0–296.3, 296.8, 296.9 | 296A-296E, 296W, 296X | F30, F31 |  |
| Schizophrenia | 295 except 295.7 | 295A–295E, 295G, 295W, 295X | F20 |  |
| Pervasive developmental disorders | - | 299A.x | F84.x |  |
| Intellectual disability | 310, 3105.x, 311, 312, 313, 314, 315 | 317, 318, 319, 758 | F70, F71, F72, F73, F78, F79, Q9 |  |
| Personality disorders | 301 | 301 | F6x, F60x |  |
| Eating disorders | - | 307B.x, 307F.x | F50 |  |
| Alcohol use disorder | 291.x, 303x | 291.x, 303x, 305A | F10 |  |
| Substance use disorder other than alcohol and tobacco | 304x | 304, 305x (except 305A and 305B), 292x | F11-F19, except F17 |  |

**Supplementary Table 4.** Groups of medication prescriptions with ATC codes

|  | **ATC** |
| --- | --- |
| Corticosteroids | D07, H02 |
| Anxiolytics | N05B |
| Hypnotics and sedatives | N05C (except for medication used for treatment of sleep disorders, see Supplementary table 3) |
| Antiepileptics | N03A, except mood stabilizers |
| Antidepressants | N06A |
| Mood stabilizers | Lithium: N05AN01 |
|  | Other mood stabilizers: N03AG01, N03AX09, N03AF01, N03AF02 |
| Antipsychotics | 1st generation: N05AA02, N05AB03, N05AD01, N05AD08, N05AF01, N05AF03, N05AF05 |
|  | 2nd generation: N05AD03, N05AE03, N05AE04, N05AE05, N05AH02, N05AH03, N05AH04, N05AX08, N05AX12, N05AX13 |
| Medication used for the treatment of ADHD | N06BA01, N06BA02, N06BA04, N06BA09, N06BA12, C02AC02 |
| Drugs used for addictive disorders (alcohol and opioid dependance) | N07BB01, N07BB03, N07BB04, N02AE01, N07BC01, N07BC02, N07BC51 |

**Abbreviations:** ADHD – attention deficit hyperactivity disorder; ATC - Anatomical Therapeutic Classification system

**Supplementary Table 5.** List of cardiovascular diseases considered as outcomes (ICD-10) and predictors (1st-degree family history, ICD-8/9/10), and corresponding ICD codes*

|  | ICD-8 | ICD-9 | ICD-10 |
| --- | --- | --- | --- |
| Ischemic heart disease | 410-414 | 410-414 | I20, I21, I22, I23, I24, I251, I252, I255, I256, I258, I259 |
| Cerebrovascular diseases and transient ischemic attack | 43 | 43 | G45, I6 |
| Thromboembolic disease | 450, 451 | 415B, 451B | I26, I80 |
| Arteriosclerosis | 440-444 | 440-444 | I70-I74 |
| Heart failure | 428 | 428 | I42, I50 |
| Arrhythmia | - | 426A, 426B, 427A, 427B, 427D, 427E, 427F, 427W | I441, I442, I46, I470, I471, I472, I48, I490, I495, I498 |

* Anatomical Therapeutic Classification (ATC) system codes used for identifying any CVD: C01A, C01B, C01D, C01E

**Supplementary Table 6.** Baseline characteristics of the study populations regarding the candidate predictors in the logistic regression model

|  | | | **Train data set** | | | **Test dataset** | | |
| --- | --- | --- | --- | --- | --- | --- | --- | --- |
|  |  |  | All  N=27,147  N (%) | With incident CVD, N=2,322  N (%) | No incident CVD, N=24,825  N (%) | All  N=6,786  N (%) | With incident CVD, N=580  N (%) | No incident CVD, N=6,206  N (%) |
| 1 | Age at follow up start (Mean, SD) | | 47.44 (12.21) | 56.96 (11.83) | 46.55 (11.86) | 47.40 (12.17) | 57.47 (11.61) | 46.45 (11.79) |
| 2 | Male sex | | 10,480 (38.60) | 1,070 (46.08) | 9,410 (37.91) | 2603 (38.36) | 259 (44.66) | 2,344 (37.77) |
| 3 | Hypertension (diagnosis or treatment) | | 3,169 (11.67) | 611 (26.31) | 2,558 (10.30) | 828 (12.20) | 174 (30.00) | 654 (10.54) |
| 4 | Diabetes (diagnosis or treatment) | | 1,518 (5.59) | 273 (11.76) | 1,245 (5.02) | 356 (5.25) | 75 (12.93) | 281 (4.53) |
| 5 | Hyperlipidemia (diagnosis or treatment) | | 2047 (7.54) | 354 (15.25) | 1,693 (6.82) | 504 (7.43) | 108 (18.62) | 396 (6.38) |
| 6 | Obesity (diagnosis) | | 1114 (4.10) | 119 (5.12) | 995 (4.01) | 290 (4.27) | 31 (5.34) | 259 (4.17) |
| 7 | Tobacco use disorder or treatment for smoking cessation | | 987 (3.64) | 123 (5.30) | 864 (3.48) | 246 (3.63) | 27 (4.66) | 219 (3.53) |
| 8 | Family history of CVD excluding individuals with missing data | | 6,358 (23.42) | 547 (23.56) | 5,811 (23.41) | 1,656 (24.40) | 161 (27.76) | 1,495 (24.09) |
|  | Individuals with missing data | | 3,651 (13.45) | 383 (16.49) | 3,268 (13.16) | 891 (13.13) | 99 (17.07) | 792 (12.76) |
| 9 | Anxiety diagnosis | | 8795 (32.40) | 698 (30.06) | 8,097 (32.62) | 2,201 (32.43) | 176 (30.34) | 2,025 (32.63) |
| 10 | Depression diagnosis | | 15,670 (57.72) | 1,412 (60.81) | 14,258 (57.43) | 3,898 (57.44) | 362 (62.41) | 3,536 (56.98) |
| 11 | ADHD diagnosis | | 1775 (6.54) | 91 (3.92) | 1,684 (6.78) | 449 (6.62) | 21 (3.62) | 428 (6.90) |
| 12 | SUD other than alcohol and tobacco use disorder diagnosis | | 3275 (12.06) | 339 (14.60) | 2,936 (11.83) | 802 (11.82) | 71 (12.24) | 731 (11.78) |
| 13 | Alcohol use disorder diagnosis | | 4463 (16.44) | 457 (19.68) | 4,006 (16.14) | 1,149 (16.93) | 122 (21.03) | 1,027 (16.55) |
| 14 | Sleep disorders diagnosis or treatment | | 16,255 (59.88) | 1,454 (62.62) | 14,801 (59.62) | 4,041 (59.55) | 355 (61.21) | 3,686 (59.39) |
| 15 | Anxiolytics | | 16,065 (59.18) | 1,378 (59.35) | 14,687 (59.16) | 4,074 (60.04) | 354 (61.03) | 3,720 (59.94) |
| 16 | Hypnotics and sedatives | | 11,637 (42.87) | 1,063 (45.78) | 10,574 (42.59) | 3,005 (44.28) | 257 (44.31) | 2,748 (44.28) |
| 17 | Antiepileptics | | 1498 (5.52) | 161 (6.93) | 1,337 (5.39) | 364 (5.36) | 39 (6.72) | 325 (5.24) |
| 18 | Antidepressants | | 20,017 (73.74) | 1,627 (70.07) | 18,390 (74.08) | 5,036 (74.21) | 413 (71.21) | 4,623 (74.49) |
| 19 | Mood stabilizers | | 19,596 (72.18) | 1,714 (73.82) | 17,882 (72.03) | 4,930 (72.65) | 425 (73.28) | 4,505 (72.59) |
| 20 | Antipsychotics | | 13,626 (50.19) | 1,266 (54.52) | 12,360 (49.79) | 3,469 (51.12) | 294 (50.69) | 3,175 (51.16) |
| 21 | Medication used for the treatment of ADHD | | 1463 (5.39) | 82 (3.53) | 1,381 (5.56) | 380 (5.60) | 18 (3.10) | 362 (5.83) |
| 22 | Drugs used for treatment of addictive disorders (alcohol and opioid dependance) | | 2575 (9.49) | 256 (11.02) | 2,319 (9.34) | 671 (9.89) | 57 (9.83) | 614 (9.89) |
| 23 | Number of hospitalizations in the last two years (Mean, SD) | | 0.91 (2.03) | 1.30 (2.76) | 0.88 (1.94) | 0.90 (1.88) | 1.17 (2.45) | 0.87 (1.81) |
| 24 | Low educational attainment excluding those with missing data | | 4646 (17.11) | 567 (24.42) | 4,079 (16.43) | 1,163 (17.14) | 144 (24.83) | 1,019 (16.42) |
|  | Individuals with missing data | | 206 (0.76) | 13 (0.56) | 193 (0.78) | 48 (0.71) | 4 (0.69) | 44 (0.71) |
| 25 | Personal disposable income* | Negative (in debt) | 191 (0.70) | 14 (0.60) | 177 (0.71) | 48 (0.71) | 1 (0.17) | 47 (0.76) |
|  |  | Zero income | 146 (0.54) | 9 (0.39) | 137 (0.55) | 32 (0.47) | 5 (0.86) | 27 (0.44) |
|  |  | Low (<20^th^ percentile) | 3870 (14.26) | 355 (15.29) | 3,515 (14.16) | 949 (13.98) | 80 (13.79) | 869 (14.00) |
|  |  | Medium (20^th^-80^th^ percentile) | 20,035 (73.80) | 1,709 (73.60) | 18,326 (73.82) | 5,004 (73.74) | 426 (73.45) | 4,578 (73.77) |
|  |  | High (>80^th^ percentile) | 2905 (10.70) | 235 (10.12) | 2,670 (10.76) | 753 (11.10) | 68 (11.72) | 685 (11.04) |
| 26 | Non-Swedish country of birth | | 3624 (13.35) | 259 (11.15) | 3,365 (13.55) | 899 (13.25) | 65 (11.21) | 834 (13.44) |
| 27 | Rural area of residence | | 1987 (7.32) | 180 (7.75) | 1,807 (7.28) | 489 (7.21) | 41 (7.07) | 448 (7.22) |
| 28 | Not married/divorced/no registered partner/widow/er | | 18,216 (67.10) | 1,478 (63.65) | 16,738 (67.42) | 4,626 (68.17) | 392 (67.59) | 4,234 (68.22) |

*Personal income was entered as a continuous variable in the logistic regression model standardized for the mean personal income for each year in the general population

**Abbreviations:** CVD – cardiovascular diseases; SD – standard deviation; ADHD – attention deficit hyperactivity disorder; SUD – substance use disorder.

**Supplementary Table 7.** Characteristics of the study population in the train and test datasets regarding the frequency of the outcome cardiovascular disease (CVD)

|  | **Training data**  N=27,147  N (%) | **Testing data**  N=6,786  N (%) |
| --- | --- | --- |
| **Incident CVD: total** | **2,322 (8.55%)** | **580 (8.55)** |
| **Incident diagnosis from the NPR** | **1,859 (6.85)** | **464 (6.84)** |
| Ischemic heart disease | 299 (1.10) | 77 (1.13) |
| Cerebrovascular diseases and transient ischemic attack | 515 (1.90) | 136 (2.00) |
| Thromboembolic diseases | 391 (1.44) | 93 (1.37) |
| Heart failure | 140 (0.52) | 40 (0.59) |
| Arteriosclerosis | 130 (0.48) | 27 (0.40) |
| Arrhythmias | 384 (1.41) | 91 (1.34) |
| **Incident medication prescription from the PDR** | **380 (1.40)** | **98 (1.44)** |
| **CVD as a cause of death from the CDR** | **113 (0.42)** | **27 (0.40)** |

**Abbreviations:** NPR- the National Patient Register; PDR – the Prescribed Drug Register; CDR – the Cause of Death Register.

**Supplementary Table 8.** Grid-search parameter space for each trained model.

| **Model** | **Parameter** | **Search space** | **Increments** |
| --- | --- | --- | --- |
| Random forest | n_estimators | 10, 50 70 100 200 300 400 | - |
|  | max_depth | 4 to 50 | 2 |
|  | min_samples_split | 2 to 10 | 2 |
|  | min_samples_leaf | 1, 2, 4 | - |
| Support Vector Machine | C | 0.05 to 0.2 | - |
|  | Kernel | Linear, poly, rbf | - |
|  | Degree | 2, 3,4 | - |
|  | shrinking | True, False |  |
|  | Gamma | Scale, auto | - |
| XGBoost | n_estimators | 20 50 70 100 200 | - |
|  | reg_lambda | 0 to 10 | - |
|  | gamma | 0 to 10 | - |
|  | learning_rate | 0.05 to 0.2 | 0.05 |
|  | max_depth | 1 to 20 | 2 |
|  | scale_pos_weight | None to 3 | - |
| Histogram-Based Gradient Boosting | Learning_rate | 0.05 to 0.2 | 0.05 |
|  | max_leaf_nodes | 1, 5, 10, 15, 20, 31 |  |
|  | max_bins | 10, 50, 100, 150, 255 |  |
|  | max_features | 0.2, 0.5, 1 |  |
|  | l2_regularization | 0, 0.1, 0.5, 0.8, 1 |  |
|  | min_samples_leaf | 1, 2, 4 |  |
|  | max_depth | None, 4 to 50 | 2 |
| Naïve Bayes | var_smoothing | 1e-4 to 1e-10 | - |

*Note.* Parameters without information on the increment had a finite range.

**Backwards stepwise procedure of additional predictors selection for the logistic regression model**

Variables were excluded consecutively based on their p-values: a variable with the highest p-value was excluded in each step of the analysis until all remaining predictors had p-values lower than 0.1.

**Predictor Estimate Standard error z value p value**

Rural residence -0.011655 0.086101 -0.135 0.892322

SUD medication -0.011979 0.085875 -0.139 0.889061

ADHD medication 0.059201 0.190693 0.310 0.756218

Depression 0.015678 0.050321 0.312 0.755368

Civil status 0.025195 0.049263 0.511 0.609036

ADHD -0.066727 0.117395 -0.568 0.569763

Alcohol use disorder 0.052482 0.064790 0.810 0.417923

Anxiety 0.049990 0.054741 0.913 0.361130

Hypnotics and sedatives 0.059496 0.049888 1.193 0.233028

Low education missing data -0.284037 0.308007 -0.922 0.356437

Income -0.123203 0.090868 -1.356 0.175145

Antidepressants -0.075289 0.054778 -1.374 0.169307

Mood stabilizers 0.082176 0.052032 1.579 0.114261

**Abbreviations:** ADHD – attention deficit hyperactivity disorder; SUD – substance use disorder.

**Supplementary Figure 1.** a) ROC curve in testing data for logistic regression, b) AURP curve in testing data for logistic regression in the test dataset

1. **b)**

**
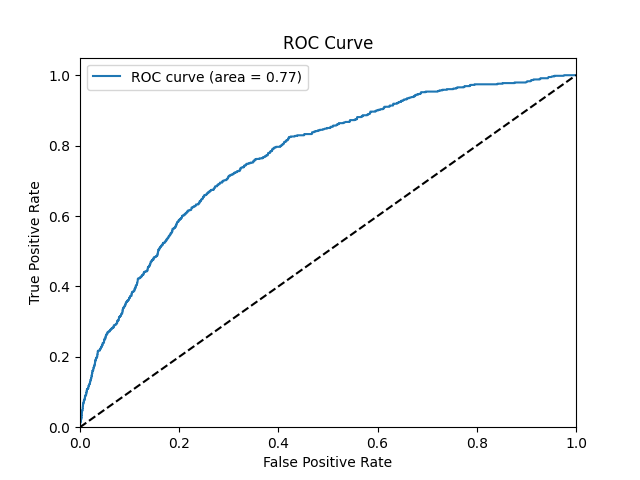

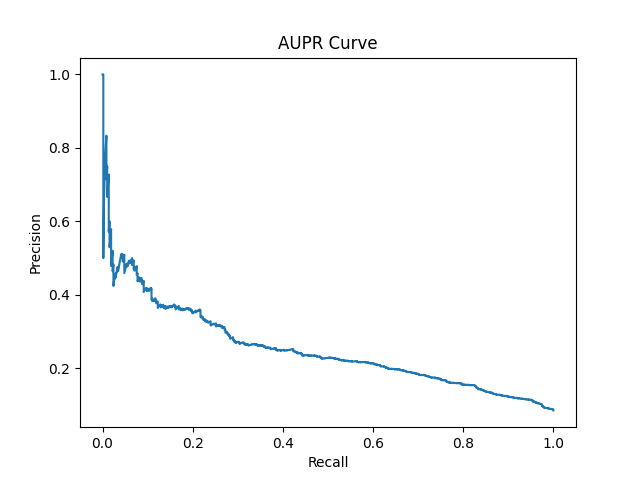
**

**Abbreviations:** ROC curve - receiver operating characteristic curve; AUPR curve - area under the precision recall curve

**Supplementary Table 9.** Performance of logistic regression containing established risk factors only (Old model) and the derived logistic regression model with additional risk factors (New model) across different subgroups in the test dataset

|  | N | Incident CVD (%) | AUC with 95% CI | | Brier score | |
| --- | --- | --- | --- | --- | --- | --- |
|  |  |  | Old model | New model | Old model | New model |
|  |  | | | | | |
| Male | 2603 | 259 (9.95) | 0.745  (0.716-0.775) | 0.747  (0.718-0.777) | 0.082 | 0.082 |
| Female | 4183 | 321 (7.67) | 0.774  (0.747-0.801) | 0.777  (0.751-0.804) | 0.064 | 0.064 |
|  |  |  |  |  |  |  |
| Younger than 50 | 4034 | 149 (3.69) | 0.678  (0.634-0.722) | 0.686  (0.644-0.729) | 0.035 | 0.035 |
| 50 and older | 2752 | 431 (15.66) | 0.671  (0.644-0.699) | 0.677  (0.650-0.704) | 0.125 | 0.124 |

**Abbreviations:** CVD - cardiovascular disease; AUC – area under the receiver operating characteristic (ROC) curve

**Post hoc analyses**

1. **Performance of the simple logistic regression model with established risk factors only separately in individuals younger than 50, and aged 50 and older in the testing data set**

| **Age group** |  | **Model in the total population** | **Retrained model** |
| --- | --- | --- | --- |
| **Younger than 50** | **AUC** | 0.678 (0.634-0.722) | 0.675 (95% CI: 0.627 - 0.718) |
|  | **Brier score** | 0.035 | 0.035 |
| **Aged 50 and older** | **AUC** | 0.671 (0.644-0.699) | 0.671 (0.644 - 0.697) |
|  | **Brier score** | 0.125 | 0.125 |

**Total population**

**coefficient SE z P>|z| [0.025 0.975]**

---------------------------------------------------------------------------------------

const -6.1609 0.118 -52.256 0.000 -6.392 -5.930

age 0.0664 0.002 32.775 0.000 0.062 0.070

male sex 0.3515 0.046 7.690 0.000 0.262 0.441

hypertension 0.4765 0.059 8.139 0.000 0.362 0.591

diabetes mellitus 0.2385 0.082 2.895 0.004 0.077 0.400

hyperlipidemia 0.0502 0.074 0.677 0.498 -0.095 0.195

obesity 0.4573 0.107 4.263 0.000 0.247 0.668

smoking 0.4177 0.104 4.023 0.000 0.214 0.621

family_history_positive 0.2083 0.055 3.776 0.000 0.100 0.316

family_history_missing 0.0582 0.064 0.906 0.365 -0.068 0.184

SE: standard error

**Younger than 50**

**coefficient SE z P>|z| [0.025 0.975]**

**---------------------------------------------------------------------------------------**

const -6.0259 0.302 -19.971 0.000 -6.617 -5.435

age 0.0628 0.007 8.652 0.000 0.049 0.077

male sex 0.2844 0.085 3.333 0.001 0.117 0.452

hypertension 0.6400 0.139 4.606 0.000 0.368 0.912

diabetes mellitus 0.2790 0.192 1.451 0.147 -0.098 0.656

hyperlipidemia 0.0882 0.191 0.462 0.644 -0.286 0.462

obesity 0.7150 0.148 4.841 0.000 0.426 1.005

smoking 0.4764 0.182 2.622 0.009 0.120 0.832

family_history_positive 0.1454 0.095 1.538 0.124 -0.040 0.331

family_history_missing 0.0858 0.131 0.654 0.513 -0.171 0.343

**Aged 50 and older**

**coefficient SE z P>|z| [0.025 0.975]**

---------------------------------------------------------------------------------------

const -6.1014 0.243 -25.109 0.000 -6.578 -5.625

age 0.0655 0.004 17.076 0.000 0.058 0.073

male sex 0.3799 0.054 6.975 0.000 0.273 0.487

hypertension 0.4482 0.064 6.989 0.000 0.322 0.574

diabetes mellitus 0.2343 0.091 2.583 0.010 0.057 0.412

hyperlipidemia 0.0544 0.080 0.679 0.497 -0.103 0.211

obesity 0.1795 0.154 1.163 0.245 -0.123 0.482

smoking 0.3845 0.126 3.046 0.002 0.137 0.632

family_history_positive 0.2345 0.068 3.441 0.001 0.101 0.368

family_history_missing 0.0565 0.074 0.765 0.444 -0.088 0.201

1. **Application of the Akaike information criterion (AIC) to select relevant predictors**

The Akaike information criterion (AIC) was applied within the simple logistic regression model by keeping fixed the established risk factors, while 30 additional risk factors were tested as candidate predictors. Additional risk factors which were kept in the model: substance use disorders, antiepileptics, antipsychotics, and the number of hospitalizations in the last two years.

Performance metrics:

Training data set – ROC AUC: 0.755 (95% CI: 0.744 - 0.766), AUPRC: 0.234 (95% CI: 0.219 - 0.251), Brier Score: 0.072 (95% CI: 0.070 - 0.074).

Testing data set – ROC AUC: 0.767 (95% CI: 0.746 - 0.786), 0.250 (95% CI: 0.220 - 0.287), Brier Score: 0.071 (95% CI: 0.066 - 0.076)

**Study Protocol**

***Design:*** Retrospective cohort study

***Data sources:*** Data on ICD-based diagnoses and ATC-based medication prescriptions will be merged from several Swedish national registers.

***Population and study period:*** We will follow individuals aged 30 and older who received a diagnosis of bipolar disorder between January 1^st^, 2007, and December 31^st^, 2018, from the date when the diagnosis of bipolar disorder was recorded until a cardiovascular disorder (CVD) diagnosis/medication prescription, emigration, death, or the end of five years, whichever came first.

***Risk factors:*** We will include relevant traditional risk factors (age, sex, history of hypertension, diabetes mellitus, obesity, hyperlipidaemia, tobacco use disorder, and family history of CVDs), psychiatric disorders (history of depression, anxiety, alcohol use disorder, substance use disorder other than alcohol and tobacco, attention-deficit/hyperactivity disorder), use of psychotropic medication, and socio-demographic variables.

***Outcomes:*** The first diagnosis of CVD after the diagnosis of bipolar disorder, recorded in the period between January 1^st^, 2007, and December 31^st^, 2014.

***Outputs:*** The main aim of the current study is to create five-year risk prediction models that will estimate the probability of obtaining a diagnosis of CVDs, with appropriate measures of predictive accuracy, and to obtain a scoring system for the calculation of cardiovascular risk at the time of the diagnosis of bipolar disorder.

**Methods**

**Data sources**

We will use data from a record linkage from multiple Swedish national registers, including the National Patient Register (NPR), Cause of Death Register (CDR), Prescribed Drug Register (PDR), and Longitudinal integration database for health insurance and labour market studies register (LISA). All diagnoses in the NPR and CDR were classified according to the International Classification of Diseases (ICD) versions 8/9/10. The Prescribed Drug Register (PDR) covers data on all dispensed medication prescriptions from July 1st, 2005, using the Anatomical Therapeutic Classification (ATC) system, with a date of prescription and dosage.

**Population and study period**

Our cohort will consist of individuals aged ≥ 30 and born between 1932 and 1984, who were diagnosed with bipolar disorder between January 1^st^, 2007, and December 31^st^, 2014, and without previous history of CVDs. The inclusion period starts on January 1^st^, 2007, to allow for enough time for medication prescriptions to be recorded (i.e., at least 18 months from the start of PDR on July 1^st^, 2005) and ends on December 31^st^ 2014. Individuals from the cohort will be followed from the date of the first diagnosis of bipolar disorder after 2007 and after age 30, with at least one previously acquired diagnosis of BD (in accordance with our definition of BD), until they acquired a diagnosis of CVD, emigrated from Sweden, died, or by the end of five years.

**Definition of bipolar disorder**

To identify patients with BD we will use a validated algorithm with high specificity from the NPR (1), where BD was defined as at least two inpatient or outpatient admissions for a core BPD diagnosis (ICD-8: 296.0-296.3, 296.8, 296.9; ICD-9: 296A-296E, 296W, 296X; ICD-10: F30, F31), with exclusion of sole diagnoses of ICD-8 296.2 (manic-depressive psychosis, depressed type) and/or ICD-9 296B (unipolar affective psychosis, melancholic form). Additionally, individuals with at least two diagnoses of schizophrenia before the start of follow up will be excluded from the cohort.

**Candidate predictors**

We selected candidate predictors a priori, based on previously established risk prediction models and relevant literature (i.e., systematic reviews and meta-analyses when available), expert opinion, availability in Swedish registry-based data and a potential for generalizability/transferability in/to other countries/contexts. Previous well-established risk prediction models for coronary heart disease include traditional risk factors such as hypertension/hypertensive therapy, body mass index (BMI), smoking, diabetes, total/LDL/HDL cholesterol (2), and family history of CVDs (i.e., first degree relative, before age 60) (3, 4). We will include proxy measures of traditional CVD risk factors, which are available in Swedish health registers: age, sex, a diagnosis/medication prescription for hypertension, diabetes mellitus (both Type I and Type II), hyperlipidaemia, obesity, and smoking; and family history (a diagnosis) of CVDs before age 60.

We will also consider psychiatric comorbidity of BD, previous use of psychotropic medication, and relevant socio-demographic factors, which are associated with an increased risk for CVDs. More specifically, the following novel risk factors will be considered: psychiatric disorders (i.e., depression, anxiety, alcohol use disorder, substance use disorder other than alcohol and tobacco, ADHD), previous use of psychotropic medication (anxiolytics, antidepressants, hypnotics and sedatives, antiepileptics, mood stabilizers, antipsychotics, stimulant and non-stimulant medication used for treatment of ADHD, and drugs used for the treatment of addictive disorders), and socio-demographic variables: educational attainment, birth country, migration status, civil status, residential area (urban/rural), employment benefits/unemployed. All relevant diagnoses, medication prescriptions, and socio-demographic information will be acquired by the date of diagnosis of bipolar disorder.

**Selection of relevant predictors**

We will use a limited backward stepwise procedure to determine whether to retain candidate predictors based on their p-values (variables with the highest p-value from the group of non-traditional risk factors will be sequentially rejected, until none of them remain with a p-value greater than 0.1) (5). Traditional risk factors will be kept in the model. With this approach we aim to retain predictors considered as traditional CVD risk factors based on clinical practice and previous studies, and to consider including novel risk factors with the final model still being easily applicable by clinician, with satisfactory face validity. Interactions between predictors will not be considered in the model.

**Outcomes**

We will include an incident diagnosis (i.e., primary or any secondary diagnosis) or treatment prescription of the following CVDs: ischemic heart disease, cerebrovascular disease, venous thrombo-embolism, arteriosclerosis, heart failure and arrhythmias, acquired after the diagnosis of BD and within a one-, two-, and five-year period. Cases of CVDs will be defined based on ICD-10 diagnostic codes from the NPR and CDR. We will include CVD medication prescriptions based on ATC codes from the PDR, to increase the coverage of cases, given that CVD conditions are often diagnosed and followed up in primary care services and are, therefore, not covered by the NPR, which only includes specialist services. Medications used as secondary prevention of CVDs will not be considered, but only those used specifically for the treatment of included CVDs.

**Statistical analysis**

We will apply standard logistic regression analysis, penalized logistic regression, random survival forest, gradient boosting machine, and survival support vector machine. For the internal validation of the models, the data will be split into a 60% training set, 20% validation set, and 20% test set. To assess the discrimination of the model, the ROC curve and c-index will be used. Hyperparameters will be tuned on the validation set. To assess the calibration of the model we will use the Brier score (6) and calibration plots (by assessing the proportion of predicted and observed events at different levels of predicted probability) (7, 8). Sensitivity, specificity, positive predictive value (PPV) and negative predictive value (NPV) will be analysed across a range of cutoff based on two high-risk thresholds of predicted probability set at 10% from a 5-year CVD risk prediction model (9) and 20% from a 10-year model (10), and percentiles of predicted probabilities (e.g., 50^th^, 75^th^, 90^th^, and 95^th^ percentile).

We will also compare the performance of the model that includes only traditional risk factors with the model which includes additional non-traditional risk factors by calculating the following measurers of incremental value: the Net Reclassification Index (NRI), which summarizes reclassification of participants when new predictors are added based on the two predefined thresholds of predicted probability; two category-free or continuous measures: the category-free NRI (11, 12), and the Integrated Discrimination Improvement (IDI) index (13), which cover all possible cut-offs or thresholds of predicted probability.

We will also test the performance of the model across the following subgroups: males and females, and in those aged younger than 50 and aged 50 and older.

**Missing data**

For all planned research questions, we will examine data missingness across variables. If less than 30% of data is missing at random, we will use multiple data imputations, with 20 imputations and five iterations through the chained equations (MICE) approach, and Rubin's rule (i.e., by accounting for the uncertainty due to missing data and the variability within and between the multiple imputations) (14). If data is not missing at random, for instance, information on family medical history or socio-demographic variables (e.g., foreign born individuals are more likely to have missing data), we will create separate variables for missingness.

**Presentation of findings and model generalizability**

The main output of the model will be a predictive probability of the occurrence of CVD after the diagnosis of bipolar disorder was recorded, within a five-year time-window. All risk factors and their estimated coefficients will be examined to create a model with face validity, and which is easy to use in practice. Considered candidate predictors should be easily assessed in the clinical setting (i.e., medical history, medical examination, psychiatric assessment, etc.).

**References from the protocol:**

1. Sellgren C, Landén M, Lichtenstein P, Hultman C, Långström N. Validity of bipolar disorder hospital discharge diagnoses: file review and multiple register linkage in Sweden. Acta Psychiatrica Scandinavica. 2011;124(6):447-53.

2. Wilson PW, D’Agostino RB, Levy D, Belanger AM, Silbershatz H, Kannel WB. Prediction of coronary heart disease using risk factor categories. Circulation. 1998;97(18):1837-47.

3. Tunstall-Pedoe H, Woodward M, Tavendale R, A'Brook R, McCluskey MK. Comparison of the prediction by 27 different factors of coronary heart disease and death in men and women of the Scottish Heart Health Study: cohort study. Bmj. 1997;315(7110):722-9.

4. Hippisley-Cox J, Coupland C, Brindle P. Development and validation of QRISK3 risk prediction algorithms to estimate future risk of cardiovascular disease: prospective cohort study. bmj. 2017;357.

5. Royston P, Sauerbrei W. Multivariable model-building: a pragmatic approach to regression anaylsis based on fractional polynomials for modelling continuous variables: John Wiley & Sons; 2008.

6. Brier GW. Verification of forecasts expressed in terms of probability. Monthly weather review. 1950;78(1):1-3.

7. Harrell FE, Jr., Lee KL, Mark DB. Multivariable prognostic models: issues in developing models, evaluating assumptions and adequacy, and measuring and reducing errors. Stat Med. 1996;15(4):361-87.

8. Harrell FE, Califf RM, Pryor DB, Lee KL, Rosati RA. Evaluating the yield of medical tests. Jama. 1982;247(18):2543-6.

9. Polonsky TS, McClelland RL, Jorgensen NW, Bild DE, Burke GL, Guerci AD, et al. Coronary artery calcium score and risk classification for coronary heart disease prediction. Jama. 2010;303(16):1610-6.

10. Grundy SM, Cleeman JI, Merz CN, Brewer HB, Jr., Clark LT, Hunninghake DB, et al. Implications of recent clinical trials for the National Cholesterol Education Program Adult Treatment Panel III guidelines. Circulation. 2004;110(2):227-39.

11. Pencina MJ, D'Agostino Sr RB, D'Agostino Jr RB, Vasan RS. Evaluating the added predictive ability of a new marker: from area under the ROC curve to reclassification and beyond. Statistics in medicine. 2008;27(2):157-72.

12. Steyerberg EW, Pencina MJ, Lingsma HF, Kattan MW, Vickers AJ, Van Calster B. Assessing the incremental value of diagnostic and prognostic markers: a review and illustration. European journal of clinical investigation. 2012;42(2):216-28.

13. Kerr KF, McClelland RL, Brown ER, Lumley T. Evaluating the incremental value of new biomarkers with integrated discrimination improvement. American journal of epidemiology. 2011;174(3):364-74.

14. Audigier V, Niang N. Clustering with missing data: which equivalent for Rubin’s rules? Advances in Data Analysis and Classification. 2023;17(3):623-57.
